# pH-Triggered Assembly of Natural Melanin Nanoparticles for Enhanced PET Imaging

**DOI:** 10.3389/fchem.2020.00755

**Published:** 2020-10-07

**Authors:** Qingyao Liu, Hanyi Fang, Yongkang Gai, Xiaoli Lan

**Affiliations:** ^1^Department of Nuclear Medicine, Union Hospital, Tongji Medical College, Huazhong University of Science and Technology, Wuhan, China; ^2^Hubei Province Key Laboratory of Molecular Imaging, Wuhan, China

**Keywords:** natural melanin nanoparticles, pH-triggered aggregation, ^68^Ga labeling, PET imaging, enhanced tumor retention

## Abstract

Natural melanin nanoplatforms have attracted attention in molecular imaging. Natural melanin can be made into small-sized nanoparticles, which penetrate tumor sites deeply, but unfortunately, the particles continue to backflow into the blood or are cleared into the surrounding tissues, leading to loss of retention within tumors. Here, we report a pH-triggered approach to aggregate natural melanin nanoparticles by introducing a hydrolysis-susceptible citraconic amide on the surface. Triggered by pH values lower than 7.0, such as the tumor acid environment, the citraconic amide moiety tended to hydrolyze abruptly, resulting in both positive and negative surface charges. The electrostatic attractions between nanoparticles drove nanoparticle aggregation, which increased accumulation in the tumor site because backflow was blocked by the increased size. Melanin nanoparticles have the natural ability to bind metal ions, which can be labeled with isotopes for nuclear medicine imaging. When the melanin nanoparticles were labeled by ^68^Ga, we observed that the pH-induced physical aggregation in tumor sites resulted in enhanced PET imaging. The pH-triggered assembly of natural melanin nanoparticles could be a practical strategy for efficient tumor targeted imaging.

## Introduction

With the continuing development of nanotechnology, there is still strong demand for the design of new nanoparticles that have the properties of biocompatibility, long circulation time, low immune response, low toxicity, and biodegradability for biomedical applications (Jiao et al., 2018; Yang et al., 2019; Ou et al., 2020). Nature has inspired scientists to mimic precise dimensional biopolymer systems that play crucial roles in the physiology of many organisms and disease processes. Great efforts have been devoted to the modification of natural nanoparticles with high applicability potential (Cormode et al., 2010; Carrera et al., 2017; Aqil et al., 2019).

Among potential nanoparticles, melanin has attracted increasing attention because of its physicochemical properties. Melanin is an endogenous pigment that is distributed widely throughout human tissues and organs such as skin, mucous membranes, retinas, gallbladder, and ovaries, making it safe for *in vivo* application (Watts et al., 1981). Recent investigations demonstrated that melanin could serve as a photothermal agent (Liu Y. et al., 2013; Chu et al., 2016) and a photoacoustic probe (Ju et al., 2016; Liu et al., 2018) because of its strong near-infrared light absorption and high photothermal conversion efficiency. Moreover, melanin is an effective drug delivery system that can load chemotherapeutic drugs with aromatic structures *via* π-π stacking and/or hydrogen binding (Zhang et al., 2015), and the drug release can be stimulated by multiple methods, including near infrared light, pH, and reactive oxygen species (Araújo et al., 2014; Wang et al., 2016; Kim et al., 2017). As the structure of melanin includes abundant carboxyl groups, amino groups, and phenolic hydroxyl groups, it can serve as a natural multi-site metal chelating agent, making it capable of complexing many metal ions under mild conditions (Kim et al., 2012; Thaira et al., 2019). Many radionuclides are metallic elements, such as ^64^Cu, ^89^Zr, ^68^Ga, ^177^Lu, and ^99m^Tc. Much effort is required to synthesize bifunctional chelators by labeling these radionuclides and optimizing the labeling conditions (Kang et al., 2015; Gai et al., 2016, 2018). Melanin may provide a facile strategy for labeling with radiometals. Cheng's group actively chelated melanin to ^64^Cu^2+^ and Fe^3+^ for PET and MRI imaging with high loading capacity and stability, indicating that melanin is a promising multimodality imaging nanoplatform (Fan et al., 2014; Hong et al., 2017).

Melanin can be made into nanoparticles with controllable sizes from a few nanometers to hundreds of nanometers (Ren et al., 2016; Amin et al., 2017; Lemaster et al., 2019). Studies have shown that small nanoparticles (<20 nm) can avoid macrophage recognition and penetrate tissues more deeply (Perrault et al., 2009; Liu C. et al., 2013). However, unfortunately, when small nanoparticles reach the tumor site, they continue to backflow into the bloodstream or are cleared into the surrounding tissues, decreasing retention within the tumor (Larsen et al., 2009; Zeng et al., 2016). Nanoparticles about 100 nm in size have been reported to have good retention but still high accumulation in the liver and pancreas before reaching the tumor, resulting in relatively low drug concentrations at the tumor site (Jain and Stylianopoulos, 2010; Albanese et al., 2012).

To overcome these limitations, we introduce a pH-triggered approach to aggregate small-sized melanin nanoparticles (pH-MNPs). The MNPs are redecorated with hydrolysis-susceptible citraconic amide, which can maintain a small size in the blood. When they reach the tumor site, spontaneous aggregation occurs in response to the tumor's acidic microenvironment. The aggregation of melanin nanoparticles cannot exceed the size of the blood vessels, and they become trapped in the extracellular matrix between cells because of their increased size, resulting in enhanced retention in the tumor site (Liu X. et al., 2013). In addition, the pH-melanin was labeled by ^68^Ga, and the *in vivo* PET imaging and biodistribution profiles of ^68^Ga-pH-MNPs were evaluated. We ascertained that the pH-triggered assembly of natural melanin nanoparticles could result in enhanced PET imaging, which could be a practical strategy for efficient tumor imaging.

## Materials and Methods

### Materials and Reagents

Melanin was purchased from Sigma-Aldrich. Methoxy polyethylene glycol amine (mPEG2000-NH_2_) was purchased from the Shanghai Aladdin Biochemical Technology Co., Ltd.

### Cell Line and Animal

H22 mouse hepatocarcinoma cells were purchased from the American Type Culture Collection (ATCC) and cultured in standard cell medium recommended by ATCC. Male BALB/c mice (6–8 weeks, 20–22 g) were provided by the animal center of Tongji Medical College (Wuhan, China). The mice were raised at an animal facility under special pathogen-free (SPF) conditions with a 12 h light/dark cycle and free access to food and water. The animal study was reviewed and approved by the Laboratory Animal Management Committee of Tongji Medical College of Huazhong University of Science and Technology.

### Preparation of PEG-Functionalized Melanin Nanoparticles (PEG-MNPs)

Thirty mg of the melanin granule was dissolved in 10 ml of NaOH (0.1 N) and sonicated for 30 min with a bath type sonicator. Then, 90 mg of mPEG2000-NH_2_ (Mw = 2,000) aqueous solution was dropped into the above aqueous solution and stirred with a magnetic stirrer. After vigorous stirring for 12 h, the mixed solution was retrieved by centrifugation (MWCO-10,000, Millipore) at 4,000 rpm for 30 min and washed several times with deionized water.

### Preparation of pH-Sensitive Melanin Nanoparticles (pH-MNPs)

Thirty mg of the melanin granule was dissolved in 10 ml of NaOH (0.1 N) and sonicated for 30 min with a bath type sonicator. Then, 90 mg of mPEG2000-NH_2_ (Mw = 2,000) and 270 μmol of ethylenediamine were added into the above aqueous solution and stirred with a magnetic stirrer. After vigorous stirring for 12 h, the mixed solution was retrieved by centrifugation (MWCO-10,000, Millipore) at 4,000 rpm for 30 min and washed several times with deionized water. Then, 200 μmol of citraconic anhydride was added into the obtained 10 ml of melanin aqueous solution (1 mg/ml of water) and the pH was adjusted to 9.0 with NaOH (0.1 N). After vigorous stirring for 12 h, mPEG and the citraconic amide modified MNPs were retrieved by centrifugation (MWCO-10,000, Millipore) at 4,000 rpm for 30 min and washed several times with deionized water.

### Characterization of Melanin Nanoparticles

The size and zeta potential of MNPs under pH 9, 7.4, and 6 were measured by a dynamic light scattering (DLS) instrument (Malvern instruments Ltd). The morphologies of MNPs were obtained under a transmission electronic microscope (TEM) at 100 kV.

### ^68^Ga^2+^ Radiolabeling

^68^GaCl_2_ was washed from a ^68^Ge/^68^Ga radionuclide generator by 4 × 1 ml high purity hydrochloric acid (HCl, 0.05 M), and we took the one with the highest radioactivity. One ml of ^68^GaCl_2_ nearly 5 mCi in 0.05 M HCl was added into 200 μl PEG-MNPs or pH-MNPs (0.5 mg/ml of MNPs), then 0.25 M NaOAc was added dropwise to adjust the pH to 4, 5, 6, 7.4, respectively and incubated at room temperature for 30 min. The radiolabeled MNPs were purified by a PD-10 column (GE Healthcare) to remove the free ^68^Ga. The final product was washed out by PBS and passed through a 0.22 μm Millipore filter into a sterile vial for *in vivo* PET imaging. The radiolabeling yield was evaluated by dividing the radioactivity of the purified radiolabeled MNPs by the total radioactivity added. The stability of ^68^Ga-labeled MNPs was determined *in vitro* by incubating in saline or human plasma at a physiologic temperature for 3 h. An aliquot of ^68^Ga-labeled MNPs was removed at 1, 2, and 3 h intervals and the radiochemical purity was determined by ITLC (TLC scanner, BIOSCAN, USA). GF254 silica gel plates were used as the stationary phase and citrate buffer (0.1 M) was used as the mobile phase.

### Cell Viability

The *in vitro* cytotoxicity of MNPs was determined in H22 mouse hepatocarcinoma cells by the CCK-8 assay. H22 cells were cultured in DMEM (GIBCO, Carlsbad, CA, USA), supplemented with 10% fetal calf serum (FCS), 2 mmol/l glutamine, 100 U/ml penicillin, and 100 μg/ml streptomycin. Cells (5,000/well) were seeded in 96-well plates with 100 μL/well medium and incubated overnight with 10% fetal bovine serum DMEM medium at 37°C and in an atmosphere of 5% CO_2_. Cells were then cultured in the medium supplemented with different doses of PEG-MNPs and pH-MNPs. The final concentrations of MNPs in the culture medium were fixed at 100, 50, 25, 10, and 5 μg/ml, untreated cells were used as the control (with 100% cell viability), and the medium without cells was used as the blank. After treatment for 24 and 48 h, respectively, the medium was removed and DMEM medium containing 10% CCK-8 was added. After incubation for 30 min at 37°C, the absorbance at 450 nm was measured by using an automatic enzyme standard instrument (Bio-Rad iMark).

### Subcutaneous Tumor Models

The H22 cells were maintained in the ascitic form by sequential passages into the peritoneal cavities of BALB/c mice, by weekly intraperitoneally (i.p.) transplanting 1 × 10^7^ tumor cells in 0.2 ml. The ascites were collected, diluted with sterile saline, and the cell concentration was adjusted to 1 × 10^7^/ml. The diluted solution (0.2 ml) was administered subcutaneously in the right shoulder of each mouse. When the tumors reached 0.5–0.8 cm in diameter, the tumor-bearing mice were subjected to *in vivo* PET imaging and biodistribution studies.

### Small Animal PET Imaging

Small animal PET imaging of tumor-bearing mice was performed on a microPET-CT (TransPET Discoverist 180 system, Raycan Technology Co., Ltd, Suzhou, China). ^68^Ga-labeled PEG-MNPs and ^68^Ga-labeled pH-MNPs (180.0 ± 5.0 μCi) were injected *via* the tail vein, respectively (*n* = 4). At different times after injection (1, 2, and 3 h), mice bearing H22 tumors were anesthetized with 2% isoflurane in 100% oxygen for maintenance during imaging, and placed prone near the center of the FOV of the scanner. PET/CT images were obtained with the static mode for 10 min followed by a CT scan in the normal mode. The PET images were reconstructed using the three-dimensional (3D) ordered-subsets expectation maximization (OSEM) algorithm with a voxel size of 0.5 × 0.5 × 0.5 mm^3^. CT images were reconstructed using the FDK algorithm with 256 × 256 × 256 matrix. Images were displayed with software Carimas (Turku PET Center, Turku, Finland). No background correction was performed. The radioactivity uptake in the tumor and normal tissues were calculated using a region of interest (ROI) drawn over the whole organ region and expressed as a percentage of the injected radioactive dose per gram of tissue (% ID/g).

### Biodistribution Studies

The biodistribution studies were performed in H22 tumor-bearing BALB/c mice (6–8 weeks), weighing 20–22 g, which were randomly divided into six groups (five mice per group). ^68^Ga-labeled PEG-MNPs and ^68^Ga-labeled pH-MNPs were intravenously injected through a tail vein and the mice were sacrificed at 1, 2, and 3 h intervals. The blood and organs of interest (e.g., brain, heart, lungs, liver, spleen, kidneys, stomach, small intestine, large intestine, muscle, bones, and tumor) were harvested, then weighed and measured using an automated gamma counter (2470 WIZARD, PerkinElmer, Norwalk CT, USA). The amount of radioactivity in each tissue sample was reported as the percentage of the injected dose per gram of tissue (%ID/g).

### Statistical Analysis

Quantitative data are expressed as means ± standard deviation (SD). Means were compared using Student's *t-*test (two-tailed) with a *P*-value <0.05 indicating significance.

## Result and Discussion

### Preparation and Characterization of pH-MNPs

**Figure 1:**
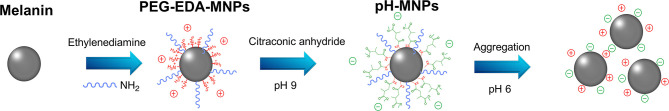
A schematic illustration of the preparation process of the pH-MNPs.

The design and synthetic procedures of pH-MNPs are schematically illustrated in Figure 1. Firstly, the natural melanin was modified with mPEG-NH_2_ and ethylenediamine to provide many terminal amine groups on the surface. Then, the primary amine groups were reacted with citraconic anhydride to form amide bonds (Figure 1). The citraconic amide moiety on the surface is selectively hydrolysis-susceptible in mildly acidic environments. Under neutral and alkaline conditions, the citraconic amide bonds are stable and maintain negative charges. Triggered by pH values lower than 7.0, such as those present in tumor tissues that are often rendered acidic by hypoxia, the citraconic amide moiety tended to hydrolyze abruptly, resulting in both positive and negative surface charges as its terminal group changed from a carboxylate anion to a protonated amine group (Nam et al., 2009). The electrostatic attraction between nanoparticles drove nanoparticle aggregation (Supplementary Figure 1). The steric effect of mPEG may have hindered the electrostatic attraction, but the reduction of the surface modification of mPEG affected the water solubility of the MNPs. A ratio of ethylenediamine to mPEG of about 6 was reported to achieve a balance between steric hindrance and water solubility.

**Figure 2:**
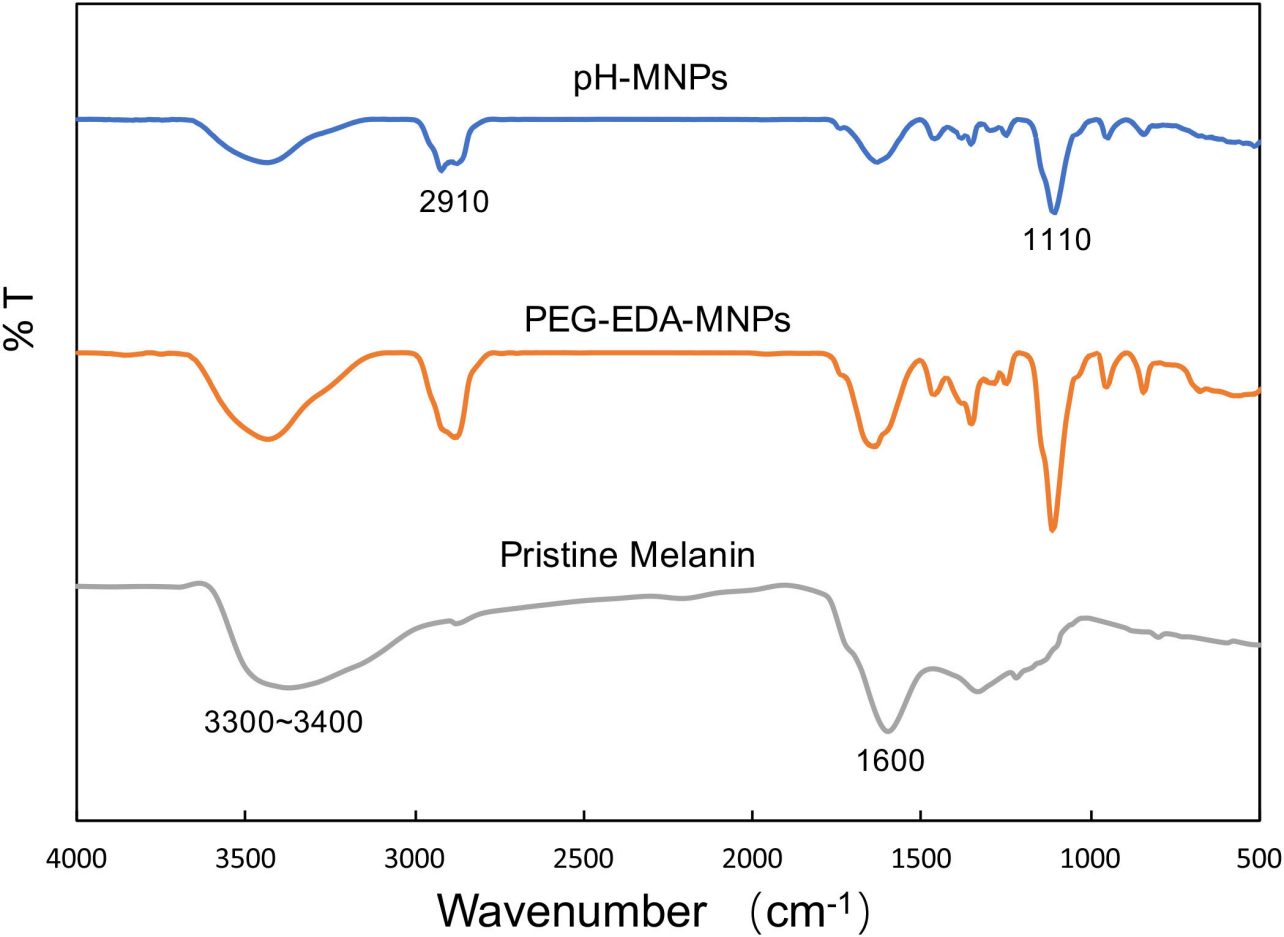
FT-IR spectra of pristine melanin, PEG-EDA-MNPs, and pH-MNPs.

**Figure 3:**
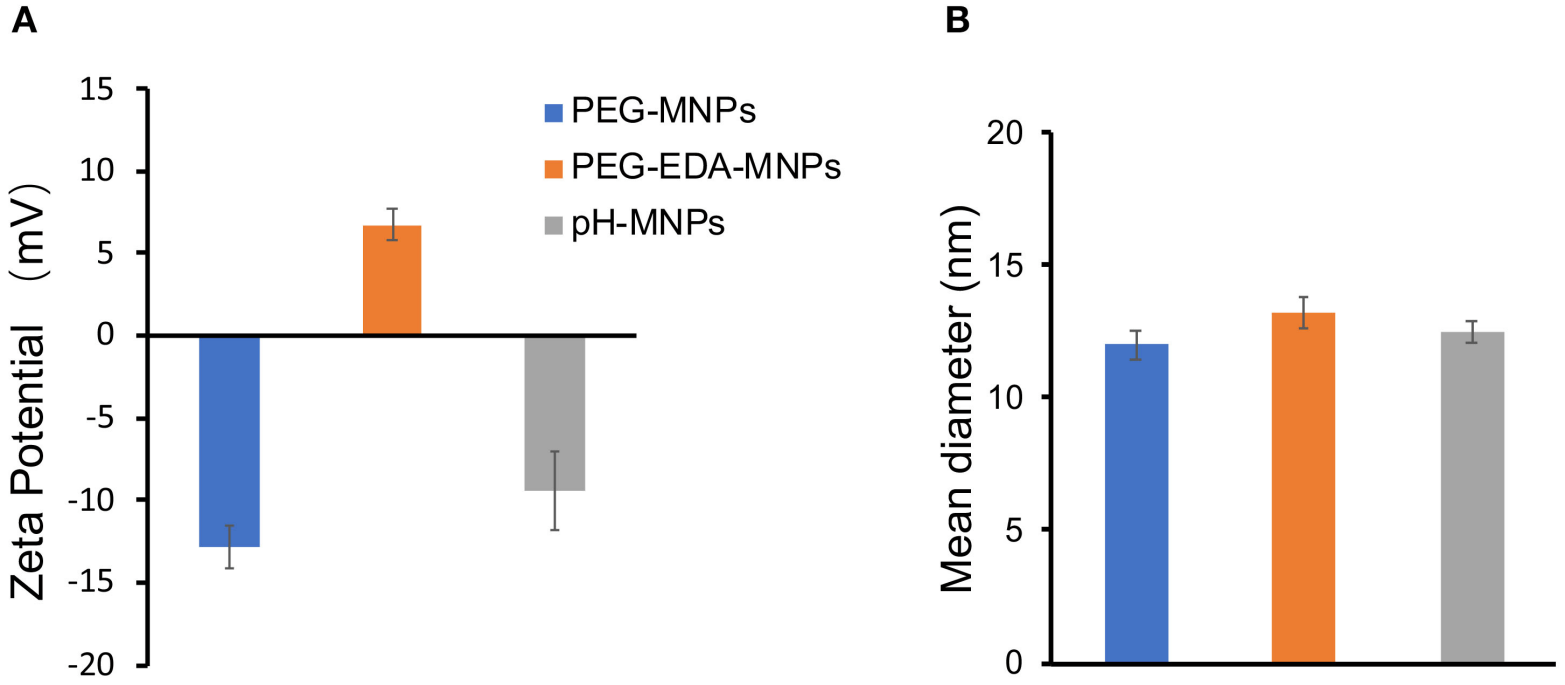
Characteristics of MNPs. **(A)** Zeta potential of PEG-MNPs, PEG-EDA-MNPs, and pH-MNPs. **(B)** Hydrodynamic size of PEG-MNPs, PEG-EDA-MNPs, and pH-MNPs. Bars represent means ± SD (*n* = 3). All of the samples are adjusted to neutral pH value by buffer solution.

The product of each step of the synthesis was measured by the zeta potential and FT-IR spectra. In the FTIR spectrum of pristine melanin, the broad and strong bands in the 3,300 ~ 3,400 cm^−1^ region were due to the -OH and -NH stretching. The characteristic peaks at 1,600 cm^−1^ were attributed to the aromatic ring C=C, C=N bending, and C=O stretching in indole and indoline structures. The FT-IR spectra detected characteristic alkyl C-H bands around 2,910 cm^−1^ and C-O-C stretching bands from PEG at 1,100 cm^−1^ after the introduction of ethylenediamine and PEG on the surface (Figure 2). Although the FT-IR spectra did not provide any additional information about the pH-MNPs, the zeta potential described a considerable change in the surface charge at each step of the surface modification (Figure 3A). Melanin itself is a negatively charged polymer, and the surface potential after the introduction of PEG remained negative (−12.8 ± 1.3 mV). After a reaction with a large amount of ethylenediamine, the surface charge changed from negative to positive (6.7 ± 0.9 mV) because of the presence of the protonated amine. The surface charge then became negative again after a reaction with citraconic anhydride, indicating successful conjugation and conversion of the amine group to a carboxylate anion. Dynamic light scattering was employed to examine the size of the MNPs after surface modification. The hydrodynamic diameters of the PEG-MNPs, PEG-EDA-MNPs, and pH-MNPs were all ~12 nm, demonstrating no significant size differences between nanoparticle type (Figure 3B).

**Figure 4:**
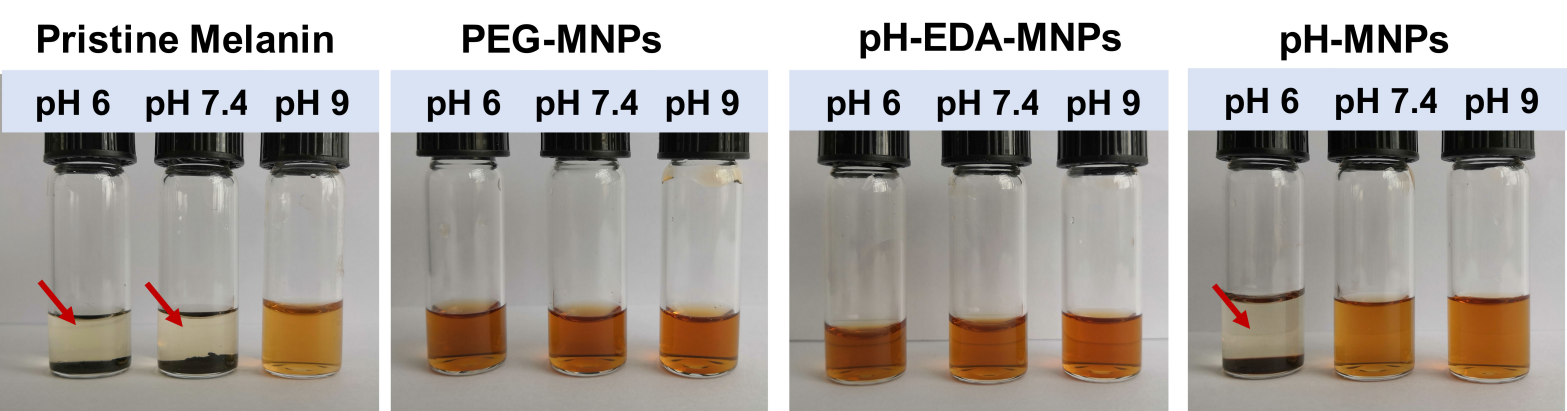
Stability of pristine melanin, PEG-MNPs, PEG-EDA-MNPs, and pH-MNPs under different pH conditions. Standing ~12 h, photos taken of all samples. The red arrow indicates precipitation at the bottom of the bottle.

To characterize pH-induced aggregation behavior in solution, we compared the stability of pristine melanin, PEG-MNPs, PEG-EDA-MNPs, and pH-MNPs under different pH conditions. As shown in Figure 4, pristine melanin only dissolved in the alkaline solution, while the PEG-MNPs and PEG-EDA-MNPs maintained good solubility in acidic, neutral, and alkaline conditions. However, pH-MNPs exhibited specific aggregation in response to acidic conditions. At pH 9 and 7.4, the solution of pH-MNPs was clear and translucent, and at a mildly acidic pH 6, flocculation and precipitation occurred. All of the photos were taken after the samples had been standing at room temperature for ~12 h, and the pH-MNPs maintained a stable precipitation state, indicating that the aggregation was irreversible after complete hydrolysis. The hydrodynamic size and zeta potential of pH-MNPs at different pH values were measured by dynamic light scattering (DLS), with PEG-MNPs as the control group. As shown in Supplementary Figure 2, the size of pH-MNPs was found to be 3,316 ± 271 nm with a wide size distribution at pH 6, while the particles showed a small size and narrow size distribution at pH 7.4 and 9. In the control group, the size of PEG-MNPs did not change and maintained at 12.2 ± 1.3 nm under different pH values. Supplementary Figure 3 showed the zeta potentials of pH-MNPs and PEG-MNPs at different pH values. At pH value of 9, the zeta potentials of pH-MNPs was −12.6 ± 1.0 mV, and the value positively shifted to −9.3 ± 1.8 mV under neutral conditions. After exposure to an acidic environment (pH 6), the surface charge of pH-MNPs shifted to a positive value (4.9 ± 0.3 mV), indicating the citraconic amide moieties had been hydrolyzed into protonated amine groups. PEG-MNPs also showed a trend in that the zeta potential shifted positively as the pH value decreased. At pH 9, the zeta potential was −16.4 ± 0.7 mV, and it became −12.8 ± 1.3 mV at pH 7.4. After exposure to pH 6 buffer, the zeta potential positively shifted to −9.3 ± 0.3 mV, but remained negative. This result was due to the protonation of phenolic and amino groups of PEG-MNPs.

**Figure 5:**
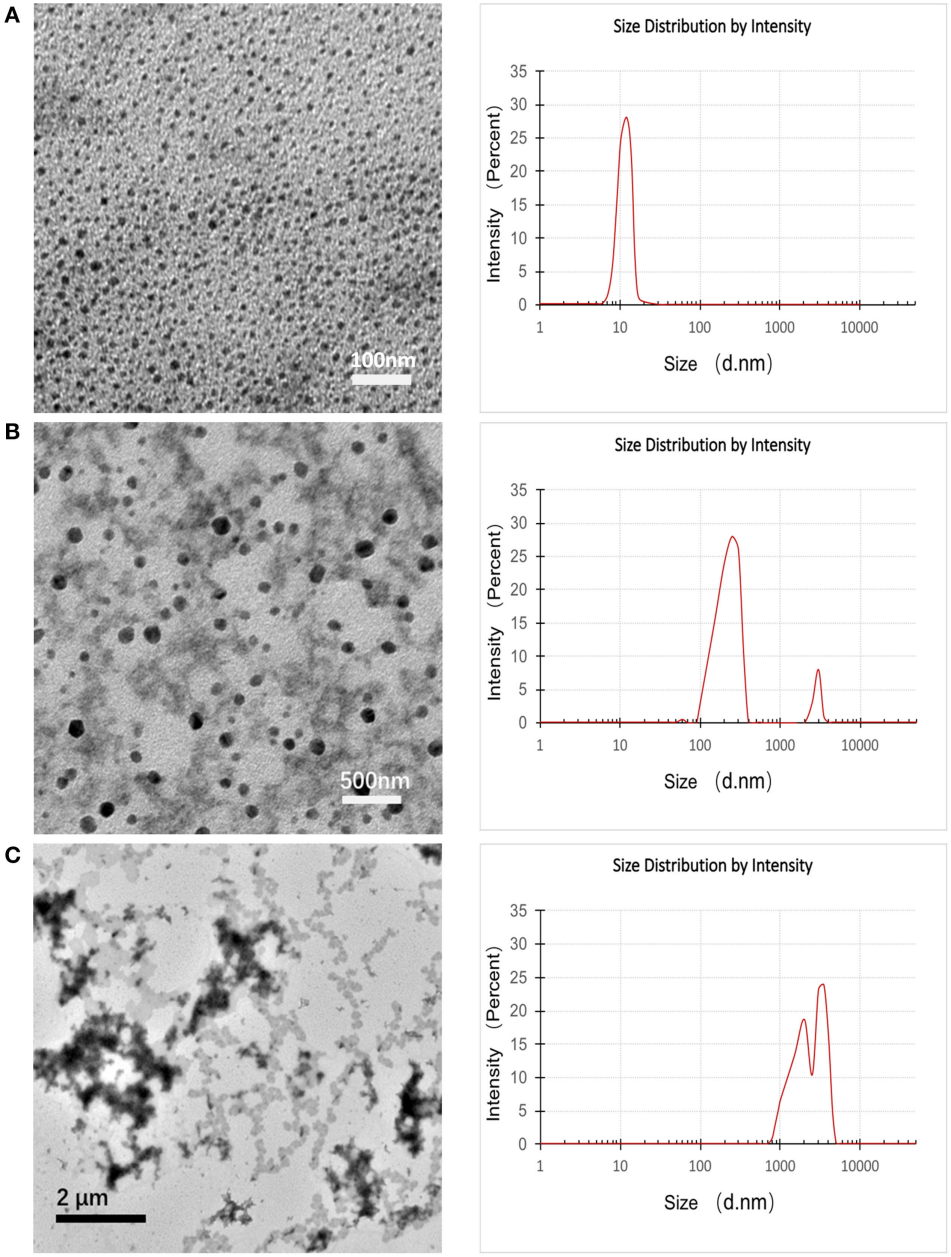
TEM images (left) and DLS images (right) of pH-MNPs in pH 6 buffer at different elapsed times of **(A)** 0, **(B)** 10, and **(C)** 120 min.

Dynamic light scattering (DLS) and transmission electron microscopy (TEM) were conducted to monitor the variation of particle size and morphology between different time points during the pH-triggering process of pH-MNPs. The TEM images in Figure 5A illustrate that the average size of the prepared pH-MNPs was nearly 10 nm with a narrow size distribution, which is consistent with the results obtained by DLS. Upon pH triggering, the agglomeration degree of pH-MNPs gradually grew, and the size to which the pH-MNPs aggregated became larger. After 10 min of exposure to an acidic environment (pH 6), the size increased to 100–160 nm with messy shapes observed by TEM, and DLS measurement showed two peaks with PDI 0.542, indicating a wide size distribution (Figure 5B). TEM measurement after 2 h of exposure confirmed the growth of some aggregates over time: the hydrodynamic size of pH-MNPs continually increased to the micron level in Figure 5C, whereas such pH-induced aggregation was not observed in PEG-MNPs (Supplementary Figure 4). These results strongly support that pH-MNPs had the ability to undergo pH-triggered aggregation. The aggregation of pH-MNPs began early (within 10 min), and flocculation occurred within 2 h. This rapid pH-response ability provides the possibility of subsequently ^68^Ga-labeling for PET imaging, which is desirable because the half-life of the ^68^Ga nuclide is only 67.7 min.

### Radiolabeling With ^68^Ga and Stability *in vitro*

**Figure 6:**
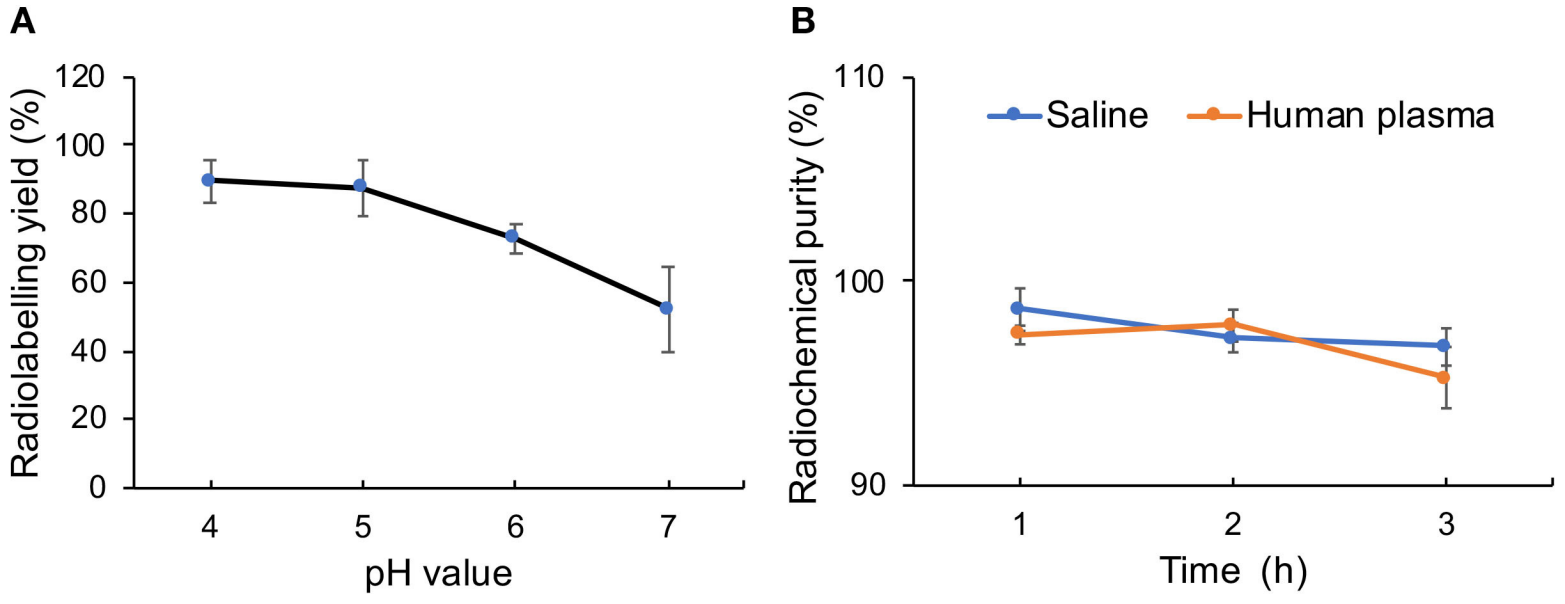
Characterization of radiolabeling with ^68^Ga. **(A)** Radiolabeling yield of ^68^Ga-pH-MNPs at different pH and **(B)** stability of ^68^Ga-labeled MNPs incubated in saline or human plasma for 1, 2, and 3 h.

Melanin has the ability to coordinate with metal ions without an additional chelator because of its inherent structure. That enables us to prepare radiometal-labeled melanin nanoparticles for molecular imaging. Furthermore, melanin can bind metal ions at a wide pH range because of different chelating sites on the molecule function at different pH ranges. Under acidic conditions, the carboxyl groups are mainly involved in binding metal ions to form complexes, whereas under alkaline conditions, the phenolic hydroxyl groups play a major role (Sarna et al., 1980). In this research, we used ^68^Ga to radiolabel pH-MNPs without any linker at different pH values. The ^68^Ga-pH-MNPs exhibited high loading capacities at pH 4 and 5 with non-decay-corrected yields of 89.6 ± 6.2 and 87.5 ± 8.3%, respectively (Figure 6A). As the pH increased, the labeling yield gradually decreased, with only 52.3 ± 12.4% yield at pH 7. Considering the acid-triggered assembly of pH-MNPs, we still tried to use ^68^Ga for labeling under neutral conditions for subsequent *in vivo* studies, but the labeling yield was not very high. The ^68^Ga-pH-MNPs were prepared under the labeling conditions of pH 7, 37°C, and 30 min incubation. After purification using a PD-10 column, the radiochemical purity of the ^68^Ga-pH-MNPs was determined by ITLC. On the ITLC plate, ^68^Ga-pH-MNPs remained close to the origin (*Rf* = 0.12), and no free ^68^Ga was observed at the solvent front (radiochemical purity: >96%; Supplementary Figure 5). The stability assay of ^68^Ga-pH-MNPs in saline solution and human plasma showed that the radiochemical purity of ^68^Ga-pH-MNPs remained above 95% throughout the 3 h incubation period, indicating excellent stability *in vitro* (Figure 6B).

### Biocompatibility of MNPs

To evaluate the *in vitro* cytotoxicity of the synthesized MNPs, CCK-8 assays were performed on H22 mouse hepatocarcinoma cells. For these assays, cultured cells were exposed to PEG-MNPs and pH-MNPs (5–100 μg/mL) for 24 and 48 h. The results showed that PEG-MNPs and pH-MNPs did not inhibit H22 cell viability at any concentration at either time point (Supplementary Figure 6), indicating that the synthesized MNPs have high biocompatibility *in vitro*.

### Small Animal PET Imaging

**Figure 7:**
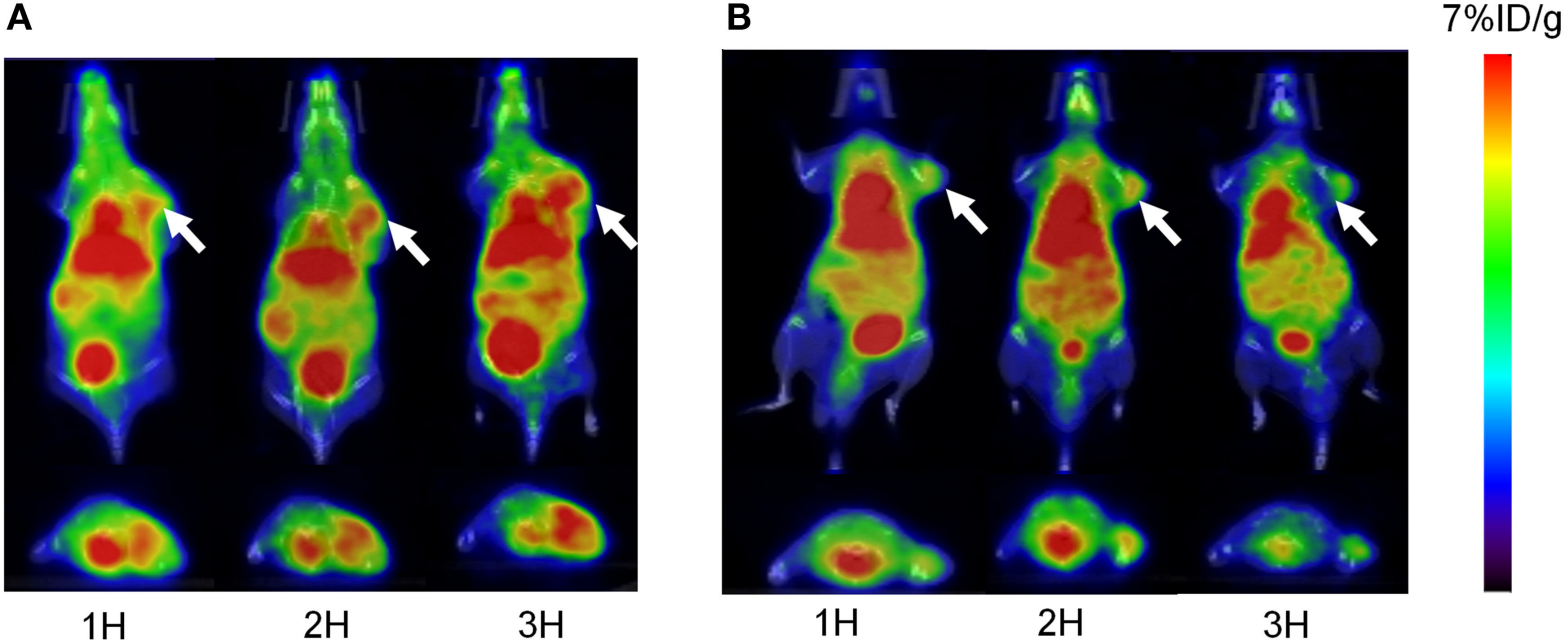
The overlaying of the PET and CT images of H22 tumors acquired at 1, 2, and 3 h after the intravenous injection of **(A)** ^68^Ga-pH-MNPs and **(B)** ^68^Ga-PEG-MNPs. Representative decay-corrected coronal and transaxial are displayed on the top and bottom respectively. The white arrow indicates tumor site.

For PET imaging, ~6.66 MBq (180 μCi) of ^68^Ga-pH-MNPs and ^68^Ga-PEG-MNPs were injected intravenously into H22 tumor-bearing mice. At different time points after injection (1, 2, and 3 h), tomographic images were acquired. Figure 7 shows representative decay-corrected whole-body images. A stronger PET signal in the tumor was observed for ^68^Ga-pH-MNPs than ^68^Ga-PEG-MNPs at all time points. The difference in tumor accumulation when ^68^Ga-PEG-MNPs are employed may be due to backflow into the bloodstream over time. In contrast, the pH-triggered aggregation of ^68^Ga-pH-MNPs, which can be trapped in tumor tissue, led to enhanced PET imaging. In addition to that within the tumor, moderate activity accumulation was found in the liver because nanoparticles are easily captured by the reticuloendothelial system. The heart was visible, perhaps because of the circulation of small-sized melanin nanoparticles in the blood. Quantitative analysis of three-dimensional regions of interest over multiple image slices revealed that the tumor uptake of ^68^Ga-pH-MNPs was up to 2.4 times higher than that of ^68^Ga-PEG-MNPs at 3 h post-injection (4.47 ± 0.73 vs. 1.87 ± 0.56% ID/g, respectively, *p* < 0.01; Supplementary Figure 7).

### Biodistribution Study

**Figure 8:**
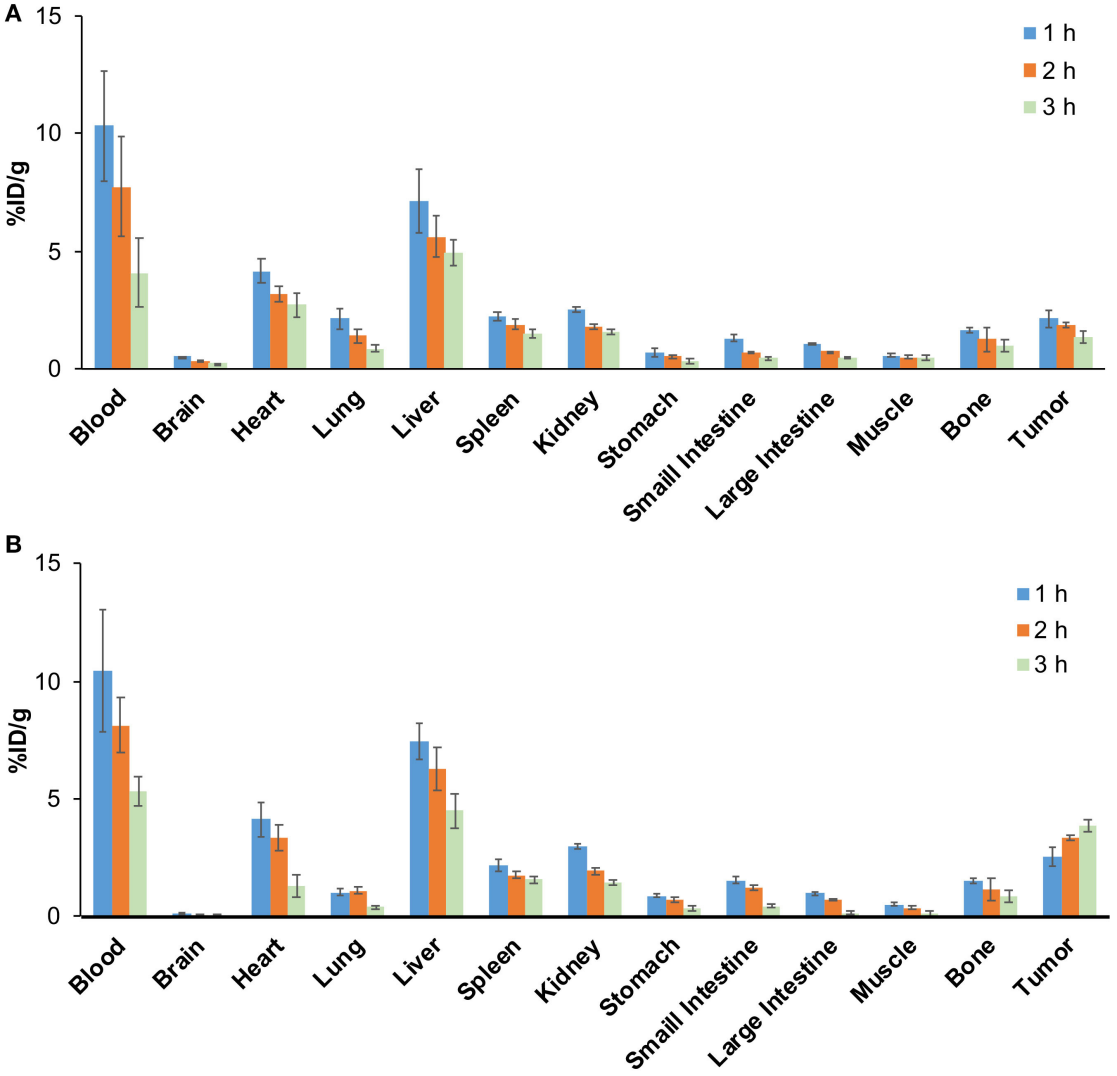
Biodistribution study of H22 tumor-bearing mice (*n* = 5) at different time points in **(A)** the ^68^Ga-PEG-MNPs group and **(B)** the ^68^Ga-pH-MNPs group.

The biodistribution results are shown in Figure 8. The radioactivity in blood gradually decreased over time, indicating that ^68^Ga-pH-MNPs were gradually cleared from circulation (Figure 8A). The liver showed the highest uptake among the tissues studied (7.47 ± 0.76% ID/g at 1 h), and then the level reduced gradually but was still prominent at 3 h post-injection (4.51 ± 0.72% ID/g). Relatively lower activity accumulation was observed in the spleen and kidney. The ^68^Ga-pH-MNPs was mainly cleared through the hepatobiliary system. The tumor uptake of ^68^Ga-pH-MNPs consistently increased, and the enhanced retention was maintained throughout all time points (2.54 ± 0.38, 3.35 ± 0.13, and 3.86 ± 0.25% ID/g at 1, 2, and 3 h p.i., respectively). In contrast, the tumor uptake of ^68^Ga-PEG-MNPs decreased from 2.14 ± 0.38% ID/g (1 h p.i.) to 1.34 ± 0.25% ID/g (3 h p.i.) (Figure 8B). The results were consistent with the PET images above. The tumor-to-muscle ratio of ^68^Ga-pH-melanin increased significantly from 4.74 ± 0.76 at 1 h p.i. to 29.30 ± 5.64 at 3 h p.i., a much greater increase than that of ^68^Ga-PEG-melanin (2.88 ± 0.74 at 3 h p.i.). However, the tumor-to-blood ratio was relatively low, probably because nanoparticles were still circulating in the blood (Supplementary Table 1). Therefore, the pH-MNPs can achieve enhanced tumor retention for PET imaging.

## Conclusions

In this work, we have successfully designed and prepared natural melanin nanoparticles that can form aggregates in response to pH changes. Under mildly acidic conditions, the pH-MNPs began to aggregate and became trapped by their increasing size, resulting in enhanced tumor retention. We also demonstrated that the pH-MNPs could be successfully radiolabeled with the ^68^Ga nuclide in a pH-neutral environment by simple mixing. The resultant ^68^Ga-pH-MNPs exhibited enhanced PET imaging, which could provide a promising strategy for molecular imaging and future clinical trials.

## Data Availability Statement

The raw data supporting the conclusions of this article will be made available by the authors, without undue reservation.

## Ethics Statement

The animal study was reviewed and approved by Laboratory Animal Management Committee of Tongji Medical College of Huazhong University of Science and Technology.

## Author Contributions

QL conceived the idea and supervised the research work overall. HF and YG contributed to the experiment methods and data analysis. QL wrote the manuscript and drew all the figures. YG came up with ideas for the manuscript. XL contributed to the revision of the paper. All authors contributed to the article and approved the submitted version.

## Conflict of Interest

The authors declare that the research was conducted in the absence of any commercial or financial relationships that could be construed as a potential conflict of interest.
