# Supplementary material for: pH-Triggered Assembly of Natural Melanin Nanoparticles for Enhanced PET Imaging
Source: Front Chem. 2020 Oct 7;8:755. doi: 10.3389/fchem.2020.00755 (PMC7579405; doi:10.3389/fchem.2020.00755)
Supplement: Supplementary file 1 [file Data_Sheet_1.docx]

Supplementary Material

# Supplementary Figures and Tables


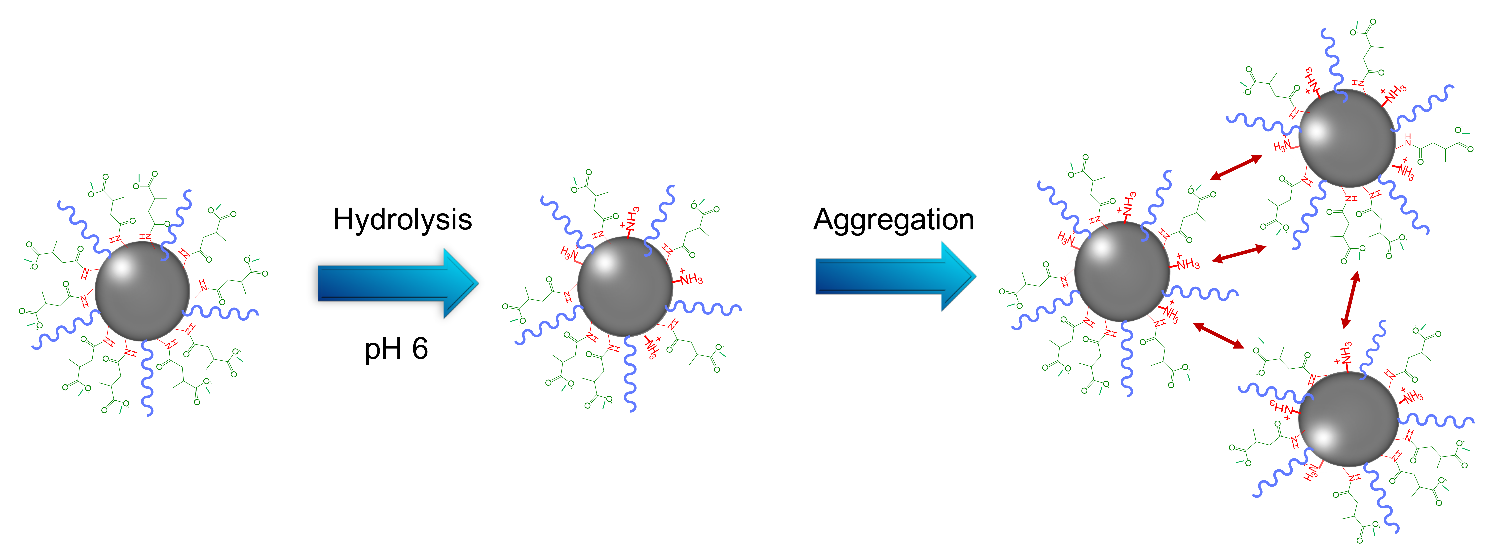


**Supplementary Figure 1.** Illustration of aggregation induced by electrostatic attraction in acidic environment.


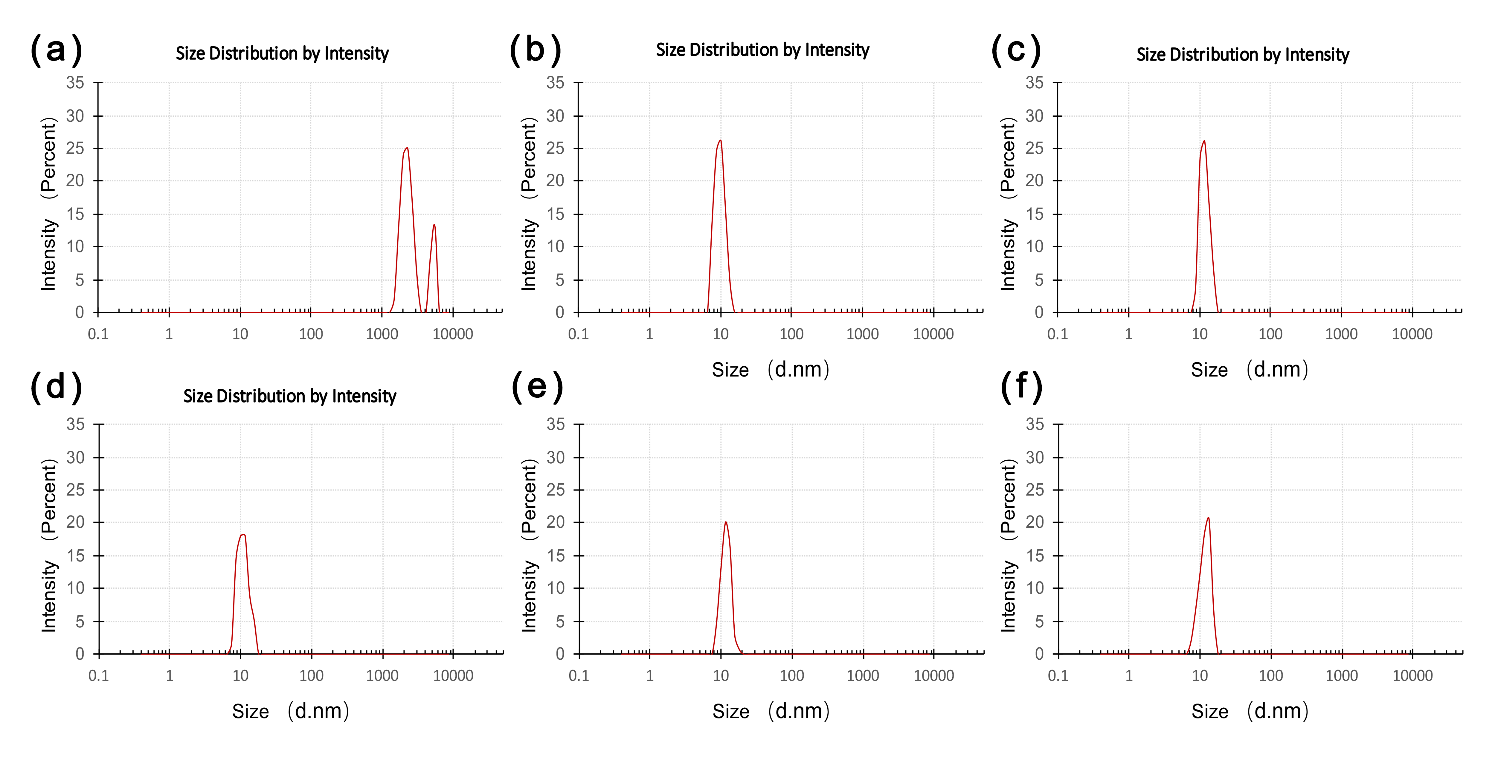


**Supplementary Figure 2**. DLS images of pH-MNPs at different pH values of (a) pH 6, (b) pH 7.4, (c) pH 9. And DLS images of PEG-MNPs at different pH values of (d) pH 6, (e) pH 7.4, (f) pH 9.


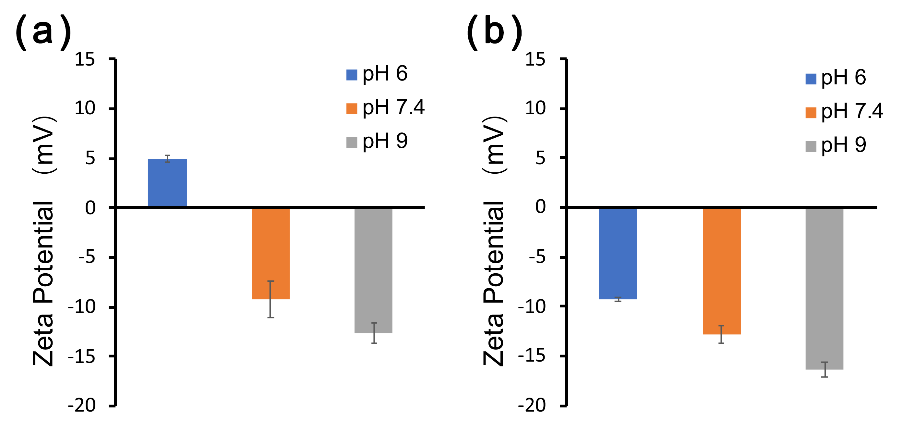


**Supplementary Figure 3.** Zeta potential of (a)pH-MNPs and (b)PEG-MNPs at different pH values.


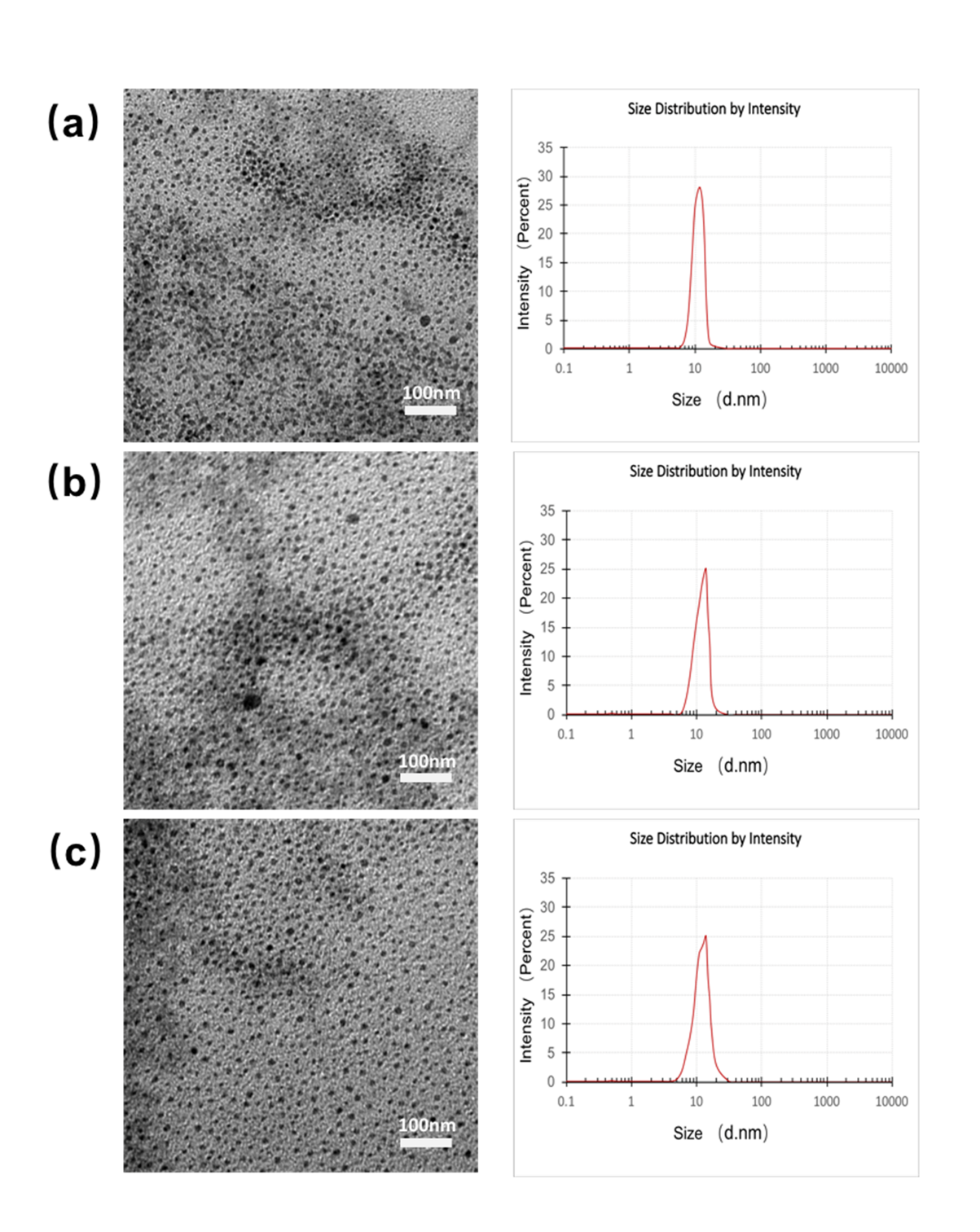


**Supplementary Figure 4.** TEM images (left) and DLS images (right) of PEG-MNPs in pH 6 buffer at different elapse time of (a) 0, (b) 10, (c) 120 min. The average size of PEG-MNPs was nearly 12 nm observed in TEM, and showed no noticeable aggregations under mild acidic condition over time.


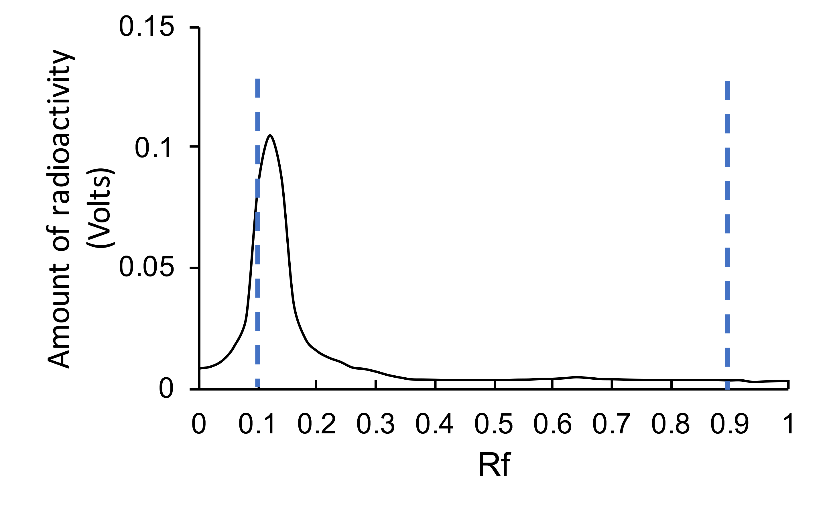


**Supplementary Figure 5.** ITLC profiles obtained for ^68^Ga-pH-MNPs. ^68^Ga-pH-MNPs remained at the origin (Rf=0.12) and no free ^68^Ga observed at the solvent front.


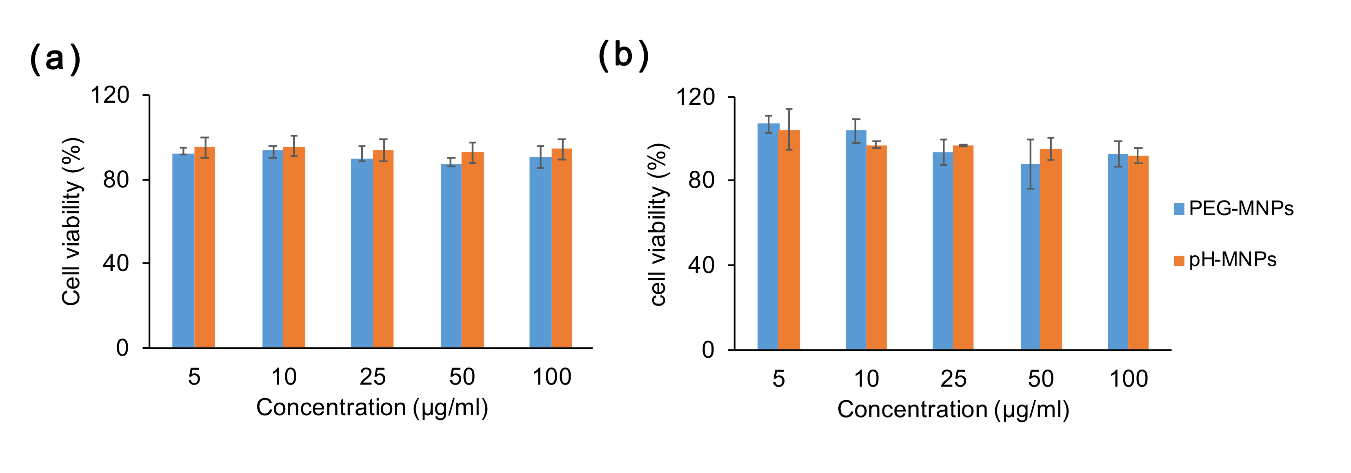


**Supplementary Figure 6.** Cell viability of H22 cells treated with 5, 10, 25, 50, 100 μg/ml PEG-MNPs or pH-MNPs with incubating time of (a) 24 h and (b) 48 h.

**
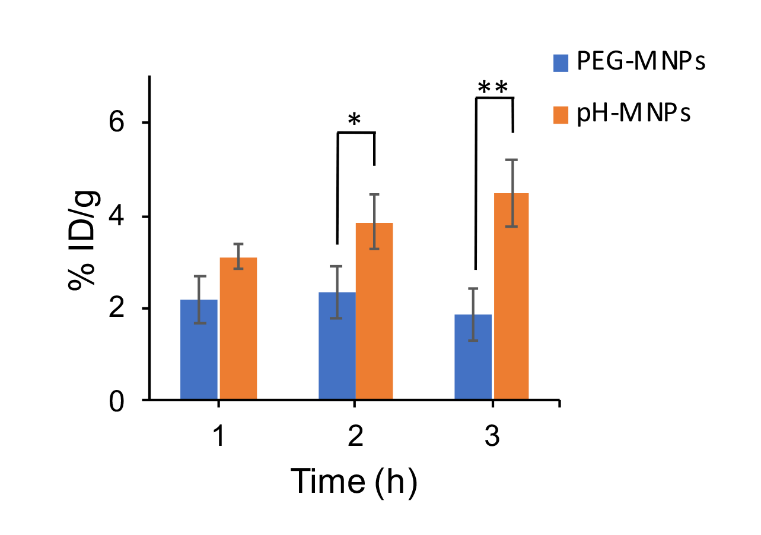
**

**Supplementary Figure 7.** The quantitative analysis of tumor site drawn by ROIs over the PET images of ^68^Ga-pH-MNPs and ^68^Ga-PEG-MNPs. Results represent the mean ± SD, * indicates p < 0.05，** indicates p < 0.01 (two tailed Student’s *t*-test).

**Supplementary** **Table 1**. The tumor-to-blood (T/B) and tumor-to-muscle (T/M) ratio of ^68^Ga-pH-MNPs and ^68^Ga-PEG-MNPs from the biodistribution study. The data values shown are means ± SD.

|  | ^68^Ga-pH-MNPs | | | ^68^Ga-PEG-MNPs | | |
| --- | --- | --- | --- | --- | --- | --- |
| Time | 1 h | 2 h | 3 h | 1 h | 2 h | 3 h |
| T/B | 0.24±0.02 | 0.41±0.04 | 0.72±0.04 | 0.21±0.02 | 0.24±0.05 | 0.33±0.04 |
| T/M | 4.74±0.76 | 8.82±1.09 | 29.30±5.64 | 3.67±0.37 | 3.70±0.57 | 2.88±0.74 |
